# Adsorption of charged anisotropic nanoparticles at oil–water interfaces†

**DOI:** 10.1039/c9na00506d

**Published:** 2019-10-07

**Authors:** Jotam Bergfreund, Qiyao Sun, Peter Fischer, Pascal Bertsch

**Affiliations:** Institute of Food Nutrition and Health, ETH Zurich 8092 Zurich Switzerland jotam.bergfreund@hest.ethz.ch pascal.bertsch@hest.ethz.ch +41 44 632 85 36

## Abstract

The adsorption of nanoparticles at fluid interfaces is of profound importance in the field of nanotechnology. Recent developments aim at pushing the boundaries beyond spherical model particles towards more complex shapes and surface chemistries, with particular interest in particles of biological origin. Here, we report on the adsorption of charged, shape-anisotropic cellulose nanocrystals (CNCs) for a wide range of oils with varying chemical structure and polarity. CNC adsorption was found to be independent of the chain length of aliphatic *n*-alkanes, but strongly dependent on oil polarity. Surface pressures decreased for more polar oils due to lower particle adsorption energies. Nanoparticles were increasingly wetted by polar oils, and interparticle Coulomb interactions across the oil phase thus increase in importance. No surface pressure was measurable and the O/W emulsification capacity ceased for the most polar octanol, suggesting limited CNC adsorption. Further, salt-induced charge screening enhanced CNC adsorption and surface coverage due to lower interparticle and particle–interface electrostatic repulsion. An empiric power law is presented which predicts the induced surface pressure of charged nanoparticles based on the specific oil–water interface tension.

## Abstract 2

Oil properties significantly affect the adsorption of charged anisotropic nanoparticles at oil–water interfaces.

## Graphical abstract

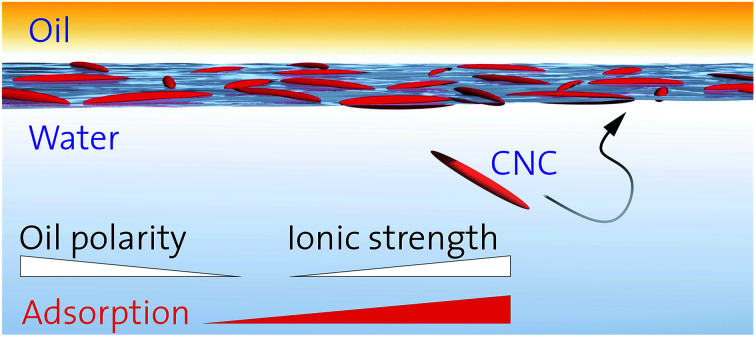

## 1 Introduction

Understanding how nanoparticles interact with fluid interfaces is crucial in various fields, including bio-imaging and drug delivery, biological membrane interactions, high-surface catalysis, or the Pickering stabilization of oil-in-water (O/W) emulsions.^1–4^ The three-phase contact line of particles at the liquid–liquid interface is mostly determined by (i) the size, shape, and surface chemistry of the nanoparticles, and (ii) the particle wetting of the two fluids.^5,6^ However, investigations of nanoparticle adsorption have long been limited to spherical model particles. Anisotropic particles induce quadrupolar interface distortions along their main axis, giving rise to attractive capillary forces for particles with overlapping distortions.^7^ At higher aspect ratio, anisotropic particles are increasingly destabilized by line tension.^8,9^ Further, nanoparticle adsorption is mostly reported for one oil, thereby neglecting the oil's wetting behavior, changes in O/W interface tension, and the oil's dielectric constant which may significantly alter nanoparticle adsorption. Here, we report on the adsorption of charged, shape-anisotropic cellulose nanocrystals (CNCs) for ten oils with a wide range of chemical structures and polarities. A generalized surface energy landscape is presented, allowing the prediction of nanoparticle adsorption depending on oil polarity.

CNCs have emerged as a biological alternative for the Pickering stabilization of O/W interfaces, paving the way for the design of biocompatible and environmentally friendly emulsions.^10,11^ The CNCs used here were obtained from wood pulp, yielding needle-like crystallites with a mean length of 79 nm and charged sulfate ester residues.^12^ Their surface charge allows the modulation of CNC interactions from repulsive to attractive by targeted salt addition, making CNCs an interesting model system to investigate the effect of particle interactions on adsorbed nanoparticle layers.^13,14^ CNC-stabilized emulsions reveal extraordinary stability against environmental influences like heat, pH, ionic strength, and even gastric conditions.^15–18^ Despite successful application in O/W emulsions, the adsorption and interactions of CNCs for different oils are mostly unknown.

## 2 Experimental section

Two inherent oil properties, namely the length of the hydrophobic backbone and oil polarity, were investigated using two sets of systematically chosen oils. For the first, linear *n*-alkanes with increasing chain length (C_8_ to C_16_) were employed. These alkanes have a constant hydrophobicity, which we define by the interface tension of a clean O/W interface, *γ*_ow_. For the second, oils with a linear C_8_-backbone and increasingly polar headgroup R were chosen, ranging from *n*-octane (R: –H), 1-chlorooctane (R: –Cl), octanal (R: =O), to 1-octanol (R: –OH). The increasing oil polarity is associated with a lower *γ*_ow_. The employed oils and their respective *γ*_ow_ values and dielectric constants are presented in Table 1. CNC adsorption kinetics were determined by changes in interface tension *γ* using profile analysis tensiometry. The dynamic surface pressure *Π* = *γ*_ow_ − *γ* was normalized with *γ*_ow_ to facilitate the comparison of different oils, *π*_norm_ = *Π*/*γ*_ow_. The employed CNCs are 79 ± 6 nm long and 5 nm in height with a linear charge density of 0.67 nm^−1^.^12,13^ The detailed Experimental section is provided in the ESI.†

**Table 1:** List of all used oils with their initial interfacial tension *γ*_0_ after purification, dielectric constant *ε* and supplier

Oil	*γ*__ow__	*ε*	Supplier
*n*-Octane	50	2	Acros (DE)
*n*-Decane	50	2	Alfa Aesar (DE)
*n*-Dodecane	51	2.03	Acros (CHN)
*n*-Tetradecane	51.5	2.05	Alfa Aesar (DE)
*n*-Hexadecane	51.5	2.08	Acros (DE)
1-Chlorooctane	35.5	5.05	Alfa Aesar (DE)
Octanal	16.7	7.61	Acros (ESP)
1-Octanol	8.7	10.3	Sigma Aldrich (USA)
Toluene	36.3	2.38	Sigma Aldrich (USA)
MCT (Myritol 318)	26	3.8–4.5	BASF (CH)

## 3 Results and discussion

**Fig. 1:**
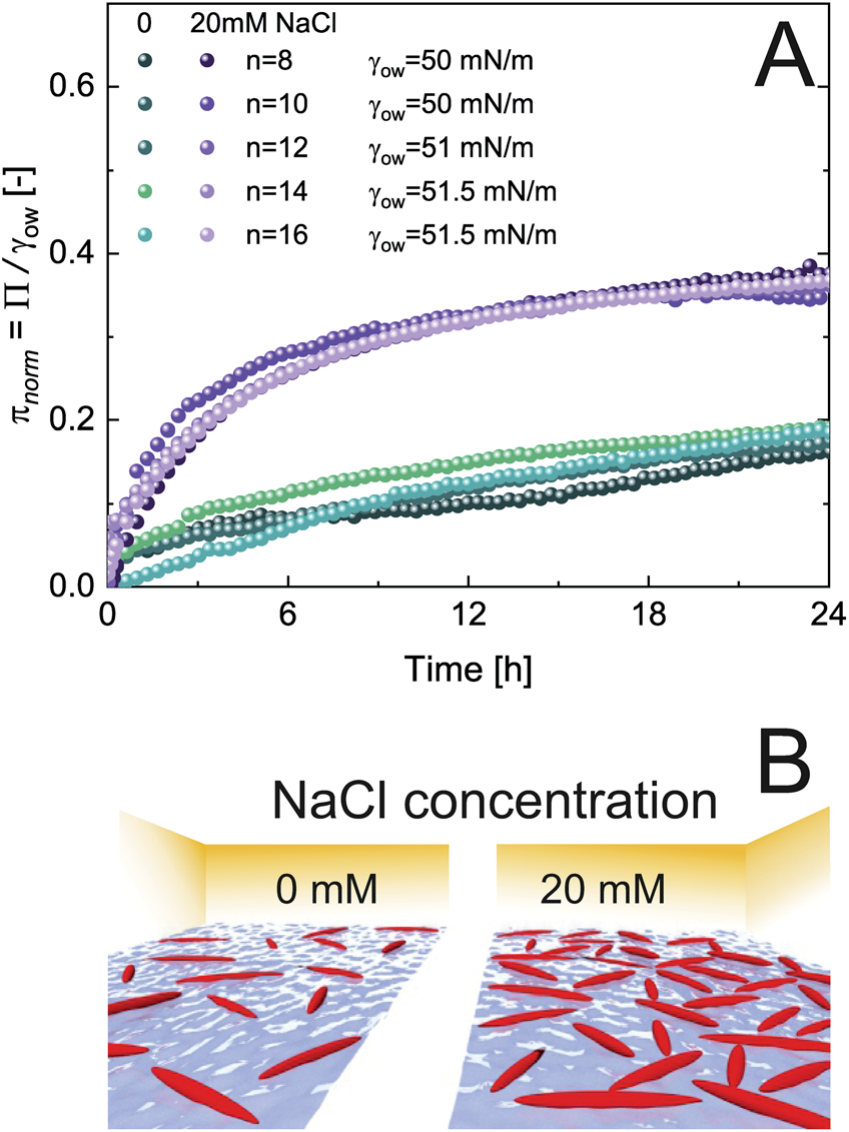
(A) Normalized surface pressure *π*_norm_ as a function of time for 0.5 wt% CNC adsorption at *n*-alkanes with increasing hydrophobic backbone length in the presence of 0 and 20 mM NaCl, determined by profile analysis tensiometry at 22 °C. (B) Schematic of the effect of charge screening on CNC surface coverage.

The adsorption of CNCs at hydrophobic *n*-alkanes with varying chain length is depicted in Fig. 1A. A bulk concentration of 0.5 wt% CNCs was chosen, which corresponds to the maximum CNC surface coverage at air–water (A/W) interfaces.^12^ CNCs adsorbed at the *n*-alkane interfaces with measurable changes in normalized surface pressure *π*_norm_ for 24 h. CNC adsorption was independent of *n*-alkane chain length and achieved a maximum *π*_norm_ of 0.23 ± 0.01 (from asymptotic fits). These adsorption kinetics are in good agreement with previous findings on CNC adsorption at the A/W interface.^12^ Nanoparticle adsorption comprises two underlying subprocesses:^19,20^ (i) diffusion limited transport to the interface and (ii) kinetically limited particle adsorption. In the case of charged nanoparticles, the kinetic adsorption barrier may be decreased by salt-induced charge screening, as demonstrated in Fig. 1A, by the addition of 20 mM NaCl. This ionic strength allows for sufficient CNC charge screening and intermolecular interactions without inducing aggregation.^12,13^ Charge screening accelerated CNC adsorption and resulted in a higher maximum *π*_norm_ of 0.38 ± 0.01. Charge screening prevents the electrostatic repulsion of CNCs in the bulk and those already adsorbed at the O/W interface. Charged nanoparticles may also be repelled from a clean interface by charged species accumulating at the interface or image charges.^21^ The adsorption-limiting effect of surface charges on adsorption was previously demonstrated for CNCs^12^ and other nanoparticles.^20,22,23^ Dugyala *et al.*^20^ employed a modified Ward–Tordai model to quantify the adsorption barrier of charged nanoparticles at fluid interfaces, confirming a decrease upon salt addition. This approach requires information on the nanoparticle adsorption energy or surface coverage, which is not provided in the present case as addressed in detail below. The higher *π*_norm_ further suggests an increased surface coverage upon salt addition. Surface charges induce repulsive capillary forces within adsorbed particles which limit their packing density.^24^ Hence, charge screening accelerates adsorption and facilitates a higher surface coverage, as schematically illustrated in Fig. 1B. The higher surface coverage may also promote attractive capillary forces as the quadrupolar interface distortions induced by anisotropic particles increasingly overlap.^7^ Interestingly, in contrast to CNC adsorption at the A/W interface, no lag phase was observed at O/W interfaces. This indicates that the kinetic adsorption barrier is lower at O/W interfaces, probably due to enhanced wetting of CNCs by oil in contrast to air. The adsorption of CNCs at the A/W interface is shown in Fig. S1A† for comparison. Adsorption independent of *n*-alkane chain length is in contrast to findings on protein adsorption, indicating a discrepancy in the adsorption behavior of rigid nanoparticles and proteins which undergo structural changes upon adsorption.^25^

**Fig. 2:**
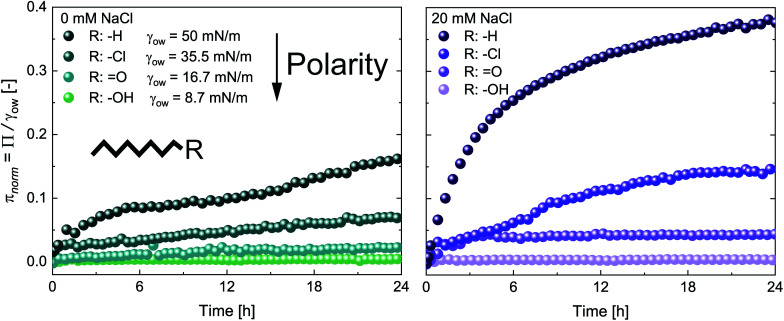
Normalized surface pressure *π*_norm_ as a function of time for 0.5 wt% CNC adsorption at oils with increasing polarity (*n*-octane, octanal, 1-chlorooctane, and 1-octanol) in the presence of (A) 0 mM and (B) 20 mM NaCl, determined by profile analysis tensiometry at 22 °C.

Fig. 2A shows the adsorption of 0.5 wt% CNCs at oils with a constant C_8_ backbone but varying polarity, revealing a good correlation between oil polarity and *π*_norm_. The surface pressure steadily decreased with increasing oil polarity, with no measurable *Π* for the most polar 1-octanol. In agreement with the findings for *n*-alkanes, CNC adsorption was enhanced by the addition of 20 mM NaCl, although no measurable changes occurred for octanol (Fig. 2B).

**Fig. 3:**
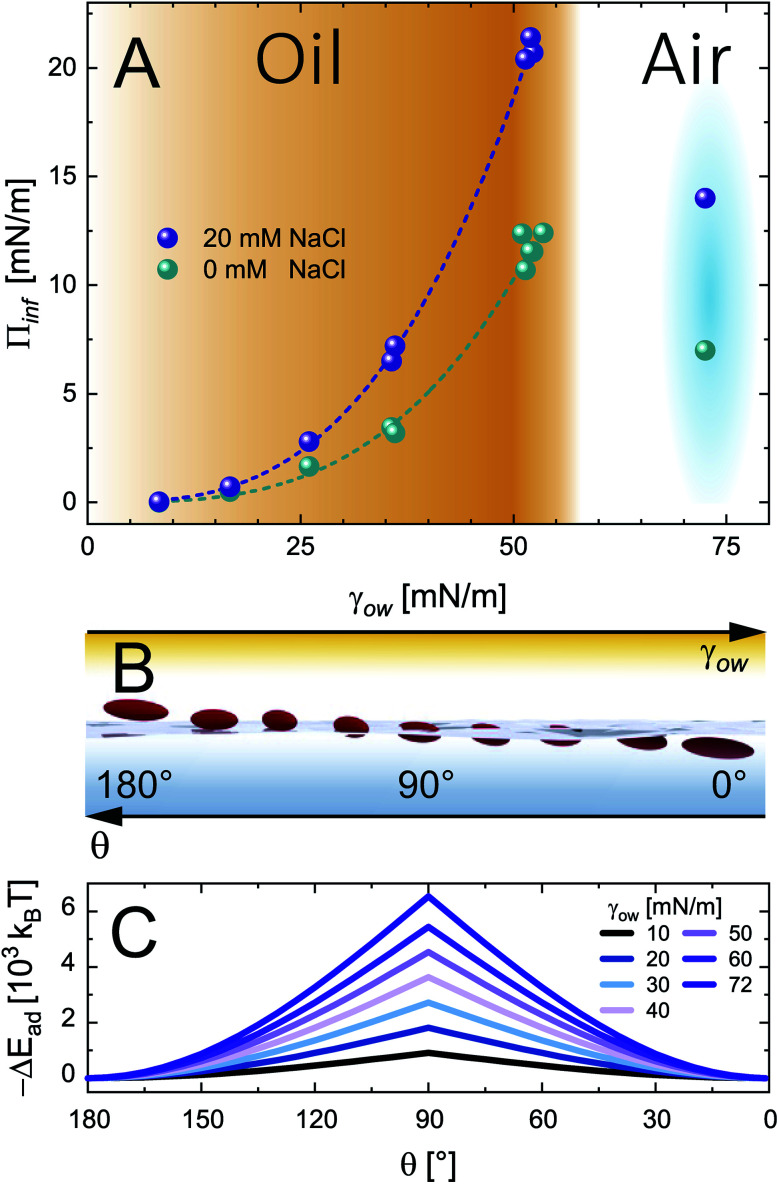
(A) Infinite surface pressure *Π*_inf_ from asymptotic fits for 0.5 wt% CNC adsorption at O/W interfaces with increasing interface tension and the A/W interface at 0 and 20 mM NaCl. The fits correspond to the power law *Π*_inf_ = *Kγ*_ow_^3^, determined by profile analysis tensiometry at 22 °C. (B) A scheme illustrating the effect of oil polarity (inverse *γ*_ow_) on the particle adsorption angle *θ*. (C) Particle adsorption energy Δ*E*_ad_ as a function of contact angle *θ* for varying interface tension *γ*_ow_ from eqn (3).

The role of O/W interface tension in CNC adsorption is apparent when plotting the fitted infinite surface pressure *Π*_inf_ as a function of *γ*_ow_, as depicted in Fig. 3A. In addition to the oils presented in Fig. 1 and 2, toluene and MCT-oil were incorporated. The respective adsorption curves are shown in Fig. S1B.† The attained *Π*_inf_ of CNCs can be generalized for all oils using a power law:

1 *Π*_inf_ = *Kγ*_ow_^3^

allowing the prediction of *Π*_inf_ only using the oil-specific O/W interface tension and a constant *K* determined by the nanoparticle surface charge and ionic strength. The measured surface pressure can be related to the adsorption energy Δ*E*_ad_ of a single CNC and the covered interfacial area fraction *η* using a displacement approach:^26^2
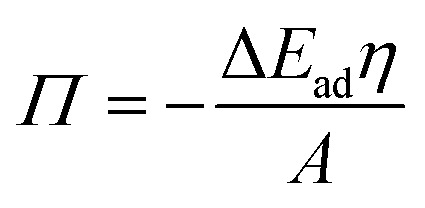
where *A* is the area occupied by one particle (370 nm^2^ from atomic force microscopy^12^). On first thought, it could be argued that *γ*_ow_ is the driving force for particle adsorption and ultimately dictates coverage. However, this is unlikely considering that CNCs have been employed to form W/W emulsions with negligible interface tension.^27^ Further, this scenario neglects particle wettability and changes in contact angle *θ* between different oils, which affects the Δ*E*_ad_ of a rod-like particle according to:^28^

3 Δ*E*_ad_ = −*Aγ*_ow_(1 − |cos *θ*|)

A scheme of varying *θ* and its effect on Δ*E*_ad_ based on eqn (3) is shown in Fig. 3B and C, respectively. The higher *Π*_inf_ observed for alkanes than for air despite lower *γ*_ow_ suggests that *θ* is closer to 90° for alkanes. For the A/W interface *θ* < 90° was reported before based on neutron reflectivity experiments.^12^ Note that anisotropic particles are destabilized by line tension with increasing aspect ratio, resulting in a lower *θ* than for spherical particles with the same surface chemistry.^8,9^ For more polar oils, CNCs are increasingly wetted by the oil, resulting in higher *θ*. Upon further immersion in the oil phase the charge environment of the aqueous phase decreases in importance. Aveyard *et al.*^29^ showed that ions at the oil/particle interface are no longer screened by the aqueous phase and contribute to long-range coulombic repulsion through the oil phase. This repulsion force *f* depends on the total charges at the oil/particle interface *q* and the dielectric constant *ε* of the oil:^30^4
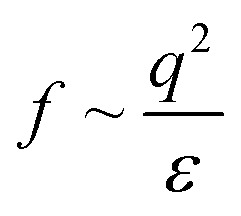


Increasing particle immersion in polar oils results in higher *q*. Although polar oils generally have a higher *ε*, it does not fully correlate with *γ*_ow_, as apparent from Table 1. Hence, with increasing particle immersion the surface coverage is limited by the oil's *ε* rather than the ionic strength in the aqueous phase, potentially impeding CNC surface coverage for polar oils. Indeed, the effect of 20 mM NaCl in the aqueous phase is less pronounced for polar oils, supporting the finding that the particles are increasingly wetted by polar oils.

The question whether *θ* can exceed 90° and particles could be primarily located in the oil phase for polar oils remains. We observed a loss in emulsification capacity for CNCs in the case of octanol. CNCs readily stabilize octane-in-water emulsions, but no stable octanol-in-water emulsions could be formed (see Fig. S2†). In the case of CNC immersion in the oil phase the formation of W/O emulsions is expected, which could not be obtained with CNCs (see Fig. S2†). Hence, *θ* increases for more polar oils but does not exceed 90°. The low *Π*_inf_ and impeded emulsification thus derive from the negligible Δ*E*_ad_ for polar oils. From the present results, it cannot be conclusively stated whether the CNC surface coverage at polar oils is limited by the low Δ*E*_ad_, or the CNCs adsorb but easily desorb again. CNCs were previously shown to accumulate at W/W interfaces with even lower Δ*E*_ad_,^27^ supporting the latter case. Our findings on impeded emulsification for polar oils are in agreement with a recent study by Bai *et al.*,^31^ who found larger emulsion droplets and impeded stability for polar oils compared to non-polar oils. There are reports on particles which can disperse in both water and oil depending on pH, ionic strength, or oil polarity, allowing the production of O/W and W/O emulsions, respectively.^32,33^ CNC crystallites have primarily hydrophilic and hydrophobic crystal planes,^34^ giving rise to speculations that CNCs must be considered amphiphilic. Our results confirm a Pickering mechanism, with CNC adsorption being driven by particle wettability.

To this point, there is a limited theoretical framework on the adsorption of charged, anisotropic nanoparticles. Further, contact angles and surface coverage of nanoparticles remain difficult to determine experimentally. Adsorption experiments for oils with varying polarity employed here could be a straightforward alternative to assess nanoparticle–oil interactions. Our results further underline that adsorption strongly depends on the employed oil. We want to emphasize this point as oil properties are often given little attention, but may strongly impede the comparability of scientific results.

## 4 Conclusions

The adsorption of charged anisotropic nanoparticles at O/W interfaces is a complex interplay of particle and interface charges, ionic strength in the aqueous phase, as well as oil properties such as particle wetting and interface tension. Although nanoparticle adsorption is energetically favorable to prevent the contact of the two phases, it may be prevented by electrostatic repulsion between particle and interface charges. This kinetic adsorption barrier can be lowered by salt-induced charge screening. We presented an empiric power law that predicts nanoparticle adsorption based on interface tension. The enhanced wetting by polar oils results in increasing particle immersion and higher *θ*. Nevertheless, the particle adsorption energy decreases due to the low interface tension. These results facilitate the prediction of nanoparticle–oil interactions and choice of the right oil–particle combination for application targeted properties.

## Conflicts of interest

There are no conflicts to declare.

## Supplementary Material

NA-001-C9NA00506D-s001

NA-001-C9NA00506D-s002
