# Supplementary material for: Adsorption of charged anisotropic nanoparticles at oil–water interfaces
Source: Nanoscale Adv. 2019 Oct 7;1(11):4308–12. doi: 10.1039/c9na00506d (PMC9419606; doi:10.1039/c9na00506d)
Supplement: NA-001-C9NA00506D-s001 [file NA-001-C9NA00506D-s001.pdf]

Electronic Supplementary Material (ESI) for Nanoscale Advances.
This journal is © The Royal Society of Chemistry 2019

Supporting Information for: Adsorption of charged anisotropic nanoparticles at oil-water interfaces

Jotam Bergfreund,* Qiyao Sun, Peter Fischer, and Pascal Bertsch*

Institute of Food Nutrition and Health, ETH Zurich, 8092 Zurich, Switzerland

E-mail: jotam.bergfreund@hest.ethz.ch; pascal.bertsch@hest.ethz.ch

Phone: +41 44 632 85 36

Contents (4 pages)

- Adsorption curves of 0.5 wt% CNC at air, toluene and MCT-oil in presence of 0 and 20 mM NaCl
- Images of CNC emulsions prepared with octane and octanol
- Experimental section

CNC adsorption at air, toluene, and MCT-oil

Figure S1A depicts the adsorption of 0.5 wt% CNC at the A/W interface. In agreement with adsorption at O/W interfaces, CNCs adsorbed within ≈ 24 hours and adsorption was accelerated and higher π_{norm} attained upon salt-induced charge screening. However, CNCs showed a lag-phase with no measurable changes in π_{norm} which was not pronounced at O/W interfaces. This could indicate a higher kinetic adsorption barrier at A/W interfaces, potentially due to enhanced wetting of CNCs by oils. The adsorption kinetics and lag phase are in good agreement with those previously reported for CNCs at the A/W interface using the Wilhelmy-plate technique.[1]

Figure S1B shows the adsorption of 0.5 wt% CNCs at toluene and MCT-oil. The fitted Π_{inf} were incorporated in Figure 3A of the main manuscript to cover a broad range of γ_{ow}. Further, MCT-oil is often used in interface science and emulsion experiments, and could be of interest for comparison with other studies.

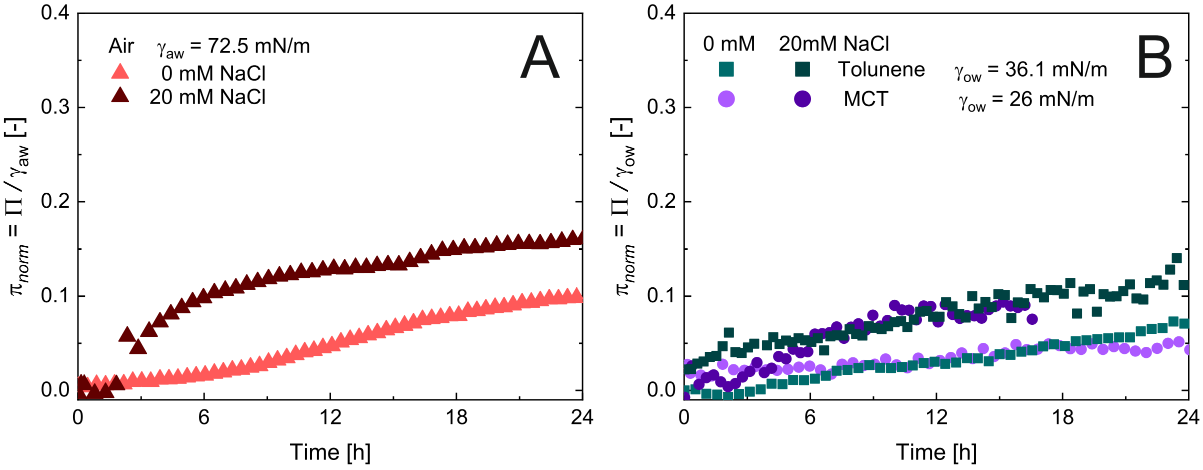

Figure S1: Normalized surface pressure π_{norm} as a function of time for 0.5 wt% CNC adsorbing at (A) air and (B) toluene and MCT-oil in presence of 0 and 20 mM NaCl. Determined by profile analysis tensiometry at 22°C.

CNC emulsification fails at polar 1-octanol

Figure S2 depicts 20 v/v% O/W emulsions prepared from n-octane and 1-octanol. A

stable O/W emulsion was formed with n-octane, whereas emulsions formed with 1-octanol showed immediate creaming of the oil phase. The emulsification of a 80 v/v% 1-octanol/water mixture was tested to check if W/O emulsion can be obtained due to CNC immersion in the polar 1-octanol. However, no stable W/O emulsion could be obtained. This suggests that emulsification is impeded due to the limited CNC adsorption energy rather than particle immersion in the oil phase. However, as specified in the main manuscript, it cannot be conclusively stated if the impeded emulsification derives from limited CNC adsorption or low stability of CNCs adsorbed at polar oils.

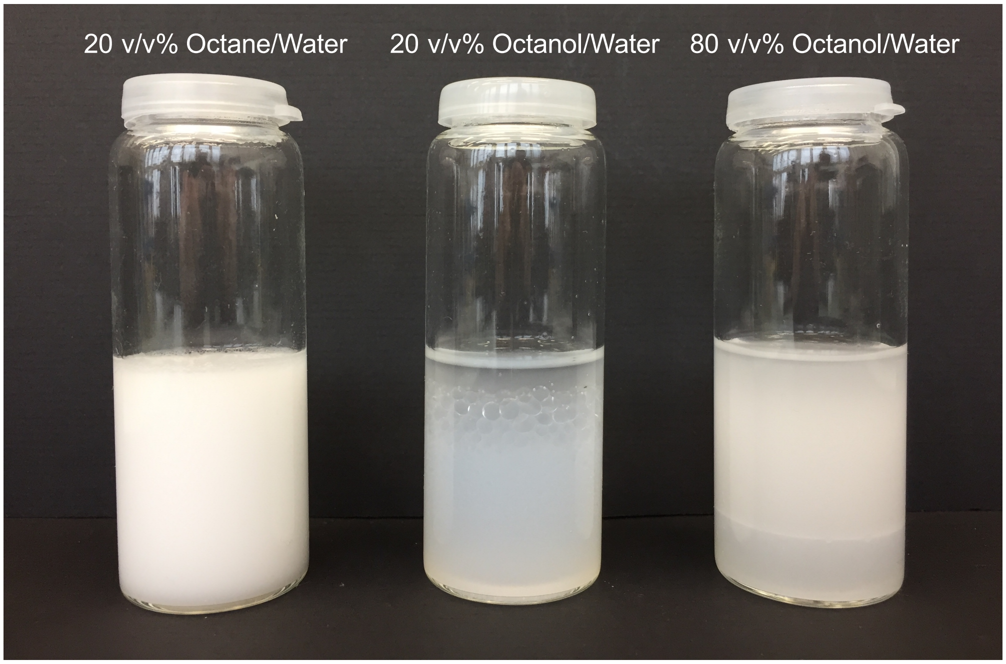

Figure S2: Images of emulsions prepared from CNC dispersions with n-octane and 1-octanol. Emulsions containing 20 v/v% O/W oil were prepared with 2 wt% CNC and 80 v/v% oil with 4 wt% CNC dispersions with 20 mM NaCl in the aqueous phase.

Experimental section

CNCs were kindly provided by CelluForce (Montreal, CA). The CNCs were dispersed in Milli-Q water (Merck Millipore, DE) and sonicated using a Hielscher UP200S ultrasonic probe (Teltow, DE) to provide full CNC deagglomeration. NaCl (Thermo Fisher, CH) was added as 1M solution and the sonication repeated.

All oils used and their suppliers, interface tension to water γ_0, and dielectric constant ϵ were compiled in Table 1 in the main manuscript. n-alkanes were purified by treatment with $FeCl_3 \cdot 6H_2O$ (Sigma-Aldrich, USA) for peroxide reduction, KOH for alcohol extraction, 40-63 μm silica gel for removal of polar contaminants, and Na_2SO_4 (all VWR Chemicals, BE) for water adsorption. Polar oils were purified with 40 g/L Florisil MgO_3Si 60-100 mesh (Fluka, CH). The oils were filtered and γ_0 checked prior to each measurement.

CNC adsorption was determined using a profile analysis tensiometer (PAT-1, Sinterface Technologies, DE). The balance between the gravitational force and the surface tension determines the droplet shape, described by the Bond number $\mathrm{Bo} = \Delta\rho \mathrm{gr}^2$, where $\Delta\rho$ is the density difference, g the gravitational acceleration, and r the radius of the droplet. An oil droplet was formed at a U-shaped capillary in a 0.5 wt% CNC dispersion and the droplet monitored by a CCD camera. The surface tension as a function of time was determined by fitting a numerical solution of the Young-Laplace equation to the droplet contour.

Emulsions were prepared from 2 wt% CNC dispersions containing 20 mM NaCl and 20 v/v% oil (n-octane and 1-octanol). The mixture was sonicated with a Hielscher UP200S at 160 W for 3 min. 80 v/v% 1-octanol was added to 4 wt% CNC dispersions to check if W/O emulsions can be obtained. Resulting emulsions were photographed 30 min after emulsification.

References

(1) Bertsch, P.; Diener, M.; Adamcik, J.; Scheuble, N.; Geue, T.; Mezzenga, R.; Fischer, P. Adsorption and Interfacial Layer Structure of Unmodified Nanocrystalline Cellulose at Air/Water Interfaces. *Langmuir* **2018**, *34*, 15195–15202.
