# Supplementary figures and images for: Adsorption of charged anisotropic nanoparticles at oil–water interfaces
Source: Nanoscale Adv. 2019 Oct 7;1(11):4308–12. doi: 10.1039/c9na00506d (PMC9419606; doi:10.1039/c9na00506d)

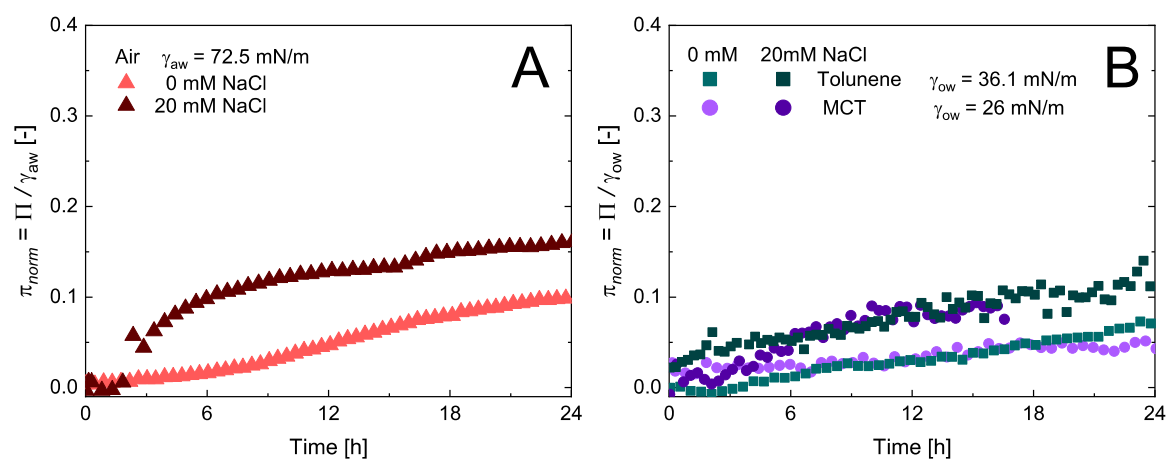

20 v/v% Octane/Water

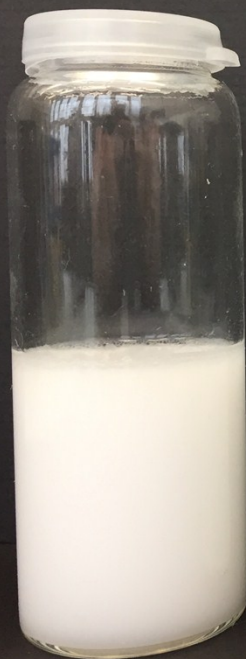

20 v/v% Octanol/Water

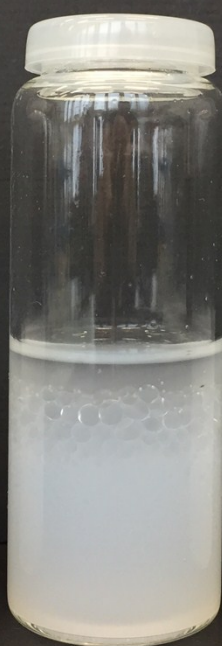

80 v/v% Octanol/Water

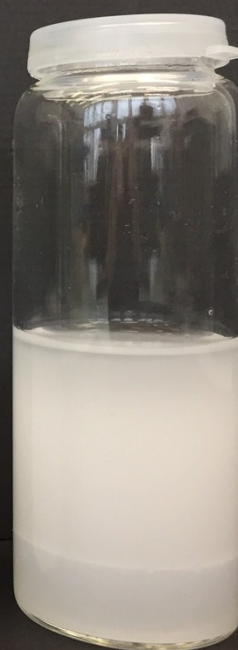

Supplement: NA-001-C9NA00506D-s002 [file NA-001-C9NA00506D-s002.pdf]
